# What works in 21st century skills education in sub-Saharan Africa: a systematic review

**DOI:** 10.3389/fpsyg.2025.1619154

**Published:** 2025-11-10

**Authors:** Stella Rose Akongo, Martin Ariapa, Mauro Giacomazzi

**Affiliations:** ALiVE Programme, Luigi Giussani Foundation, Kampala, Uganda

**Keywords:** life skills education, 21st century skills, socio-emotional learning, assessment of life skills, sub-Saharan Africa

## Abstract

This systematic review explores the relevance, implementation, and assessment of 21st century life skills education in sub-Saharan Africa, with a focus on socio-emotional learning, soft skills, and resilience. It identifies life skills as essential for youth development, particularly among vulnerable populations facing social and economic adversity. Despite policy recognition across sub-Saharan Africa, life skills integration into education systems remains inconsistent due to infrastructural gaps, teacher preparedness, and lack of culturally relevant frameworks. The review analyses 27 intervention assessments, revealing that experiential, structured, and contextually adapted pedagogies—particularly those targeting internal domains like self-esteem and self-efficacy—yield significant psychosocial and educational outcomes. It also highlights a critical gap in validated, context-sensitive assessment tools, with most relying on Western self-report measures. Community and school-based programs showed positive outcomes, especially when grounded in participatory learning and local relevance. The study underscores the need for scalable models, systemic evaluation, and policy alignment, and advocates for strengthening teacher training and community involvement. It concludes with recommendations to enhance life skills programming through sustained, context-specific approaches and improved measurement frameworks. The findings aim to inform policymakers, educators, and practitioners in developing effective strategies for fostering youth competencies across the region.

## 1 Introduction

### 1.1 Relevance of 21st century skills in sub-Saharan Africa

Life skills, 21st century skills, socio-emotional learning (SEL), soft skills, transversal skills are just a few of the terms that literature uses to indicate the breadth of skills that are considered indispensable for equipping young people in sub-Saharan Africa with the competencies needed to navigate social and economic challenges (Care, 2024; Sajeevan et al., 2024). By fostering critical thinking, problem solving, emotional intelligence, and social connectedness, 21st century skills education plays a pivotal role in improving academic outcomes, workforce readiness, and overall well-being. Strengthening policies and scaling up evidence-based interventions is key to ensuring that all youth—particularly those who are socially vulnerable—benefit from these essential competencies (Care and Giacomazzi, 2024).

Life skills have gained significant importance in sub-Saharan African countries due to several factors. Governments in the region have recognised life skills and values as a major educational priority to enhance social wellbeing and global economic competitiveness (Giacomazzi et al., 2022a). This focus on life skills is rooted in the understanding that they are essential for youth to thrive in various environments, including school, home, and their communities (Hermens et al., 2017).

The relevance of life skills is particularly pronounced for socially vulnerable youth in sub-Saharan Africa (Care and Giacomazzi, 2024). Studies suggest that SEL programs in low-resource settings can contribute to positive youth development by improving academic achievements and reducing inequalities in both academic and social settings (Jukes and Norman, 2024). Vulnerable youth in sub-Saharan Africa often face numerous stressors in their daily lives, such as income poverty, poor family management, low housing quality, and exposure to peers involved in problem behaviour (Hermens et al., 2017). Life skills are viewed as vital tools to help youth navigate these challenges and improve their overall wellbeing, academic performance, and future job satisfaction (Hermens et al., 2017). For example, young vulnerable people who develop strong emotional skills—such as managing stress, resolving conflicts constructively, and staying motivated—are better equipped to maintain their wellbeing, achieve higher academic results, and experience greater satisfaction in their future careers (Hermens et al., 2017). Cognitive skills, such as self-regulation and decision making, serve as protective factors against the stressors they encounter (Hermens et al., 2017). Social skills are essential in helping these youth decrease social disconnectedness, a major indicator of social vulnerability (Hermens et al., 2017).

The growing recognition of 21st century skills as a key educational priority in sub-Saharan Africa further underscores the relevance of investigating these skills and better understanding how to assess and nurture them (Care and Giacomazzi, 2024).

Governments in sub-Saharan Africa have recognised the importance of incorporating 21st century skills into their curricula (Giacomazzi et al., 2022a). However, implementation faces numerous obstacles, including ill-adapted content, inadequate infrastructure, and lack of teacher preparedness (Jared et al., 2016).

Teacher education programs need to evolve to effectively integrate 21st century skills, as current practices often fall short (Ntuli et al., 2016; Kausar and Ajmal, 2024); challenges persist in applying theoretical knowledge to pedagogical practices (Kausar and Ajmal, 2024). To overcome these obstacles, teacher education programs should focus on targeted interventions, problem-based learning, project-based learning, peer-based learning, and technology-based teaching-learning approaches (Almazroa and Alotaibi, 2023). Additionally, programs should emphasise personal development of 21st century skills in preservice teachers before application in educational contexts (Brassard et al., 2021).

### 1.2 Lack of evidence on effective 21st century skills programs

Despite the recognised importance of 21st century skills, there is a significant gap in the literature regarding effective strategies for nurturing and assessing these skills in sub-Saharan Africa (Giacomazzi et al., 2022a). This lack of evidence can be attributed to several interconnected factors. One of the most relevant is linked to the fact that many schools and education systems in sub-Saharan Africa struggle to translate government policies on life skills into practical curricula, pedagogies, and assessment frameworks (Schweisfurth, 2011). The lack of clear implementation strategies hinders the study and evaluation of these programs in real-world educational settings. Additionally, resource constraints, teacher training deficiencies, and misalignment between policy and classroom realities exacerbate these challenges (Oketch et al., 2021).

Moreover, researchers and practitioners often apply Western frameworks of 21st century skills to the sub-Saharan African context without adequately considering their cultural relevance (Giacomazzi et al., 2022a). Studies indicate that life skills education must be adapted to local values, societal structures, and economic conditions to be effective (Giacomazzi et al., 2023). For instance, communal approaches to problem solving and interdependence, which are deeply rooted in many African societies, may not align with Western individualistic perspectives (Giacomazzi, 2021; Care and Giacomazzi, 2024).

A major barrier to advancing research on life skills effectiveness is the lack of culturally adapted assessment tools (Mugo et al., 2022, 2023; Sajeevan et al., 2024). Many existing life skills evaluation frameworks rely on self-reporting methods, which may not capture nuanced social behaviours and cognitive development in diverse cultural contexts (Kautz et al., 2017). Additionally, assessment tools developed in Western contexts may not be fully applicable to African students due to linguistic differences, differing educational expectations, and contextual variations in socialisation (Giacomazzi et al., 2022a).

The majority of life skills programs in sub-Saharan Africa lack systematic implementation, evaluation, and monitoring. Inconsistent methodologies, fragmented interventions, and weak institutional coordination contribute to a lack of robust, comparable evidence across different contexts (Brunello and Schlotter, 2011). Recent research highlights the persistent issue of donor-driven development programs, often resulting in short-term projects without sustained impact evaluation (Harris, 2023). Many programs are detached from local needs and market demands, focusing on outputs rather than long-term outcomes (Harris, 2023). Short-term project cycles often ignore the fact that meaningful change may take generations to manifest (Pratt, 2018). Evaluations tend to focus on individual-level impacts, neglecting organisational and community-level effects. To address these issues, experts recommend adopting a systems lens in evaluations, promoting community ownership and engagement (Ilesanmi and Afolabi, 2022), and incorporating sustainability planning from the project design phase (Cekan/ova, 2023).

### 1.3 Problem statement

While the development of 21st century skills has been widely recognised as essential for preparing students to navigate an increasingly complex and technology-driven world, research on the cultivation and assessment of these skills in sub-Saharan Africa remains fragmented and inconsistent, limiting the ability of educators and policymakers to implement effective strategies (Giacomazzi et al., 2022a). Research on 21st century skills education in sub-Saharan Africa faces numerous challenges, including high attrition rates in longitudinal studies, funding constraints, and ethical concerns (Lema et al., 2010). These barriers contribute to small sample sizes and potential bias in findings (Zickafoose et al., 2024). Existing studies on life skills interventions often lack robust comparison groups and experimental controls, making it challenging to determine causality (Mwale and Muula, 2017; Nasheeda et al., 2018).

While global frameworks provide a conceptual foundation for 21st century skills assessment (Greiff and Kyllonen, 2016), their applicability in sub-Saharan African contexts remains underexplored (Fontana et al., 2022; Giacomazzi et al., 2022b). Variability in educational settings, pedagogical approaches, and resource availability further complicates efforts to synthesise best practices (Chalkiadaki, 2018; Nasheeda et al., 2018). Teacher preparation and professional development remain key challenges, as integrating these skills into curricula often requires project-based learning and advanced digital tools that may not be readily available or effectively utilised (González-Salamanca et al., 2020).

Furthermore, while some studies highlight the potential of targeted interventions to improve teacher readiness (Almazroa and Alotaibi, 2023), the sustainability and scalability of these approaches in sub-Saharan Africa remain uncertain. Persistent challenges in implementation—including disparities in infrastructure, teacher training gaps, and policy misalignment—underscore the need for a systematic review that synthesises existing evidence and identifies practical pathways for nurturing and assessing 21st century skills in the region.

In this context, the Regional Education Learning Initiative (RELI), through a network of civil society organisations working closely with academia and governments, is bridging this gap. Through the regional Actions for Life Skills and Values in East Africa (ALiVE), RELI aims at supporting education systems to embed 21st century skills into curricula, assessment and teacher education, developing context-relevant assessments and strengthening local expertise. In the past 4 years, ALiVE has developed 4 assessment tools, trained 47 local experts who are supporting integration of life skills into curriculum and assessment, assessed over 45,000 adolescents across Kenya, Tanzania, and Uganda and engaged over 5 million government officials, policy makers, parents, and civil society/local actors.

## 2 Research questions and objectives

A comprehensive mapping of nurturing approaches and assessment tools for life skills in sub-Saharan Africa was conducted to identify evidences and promising interventions, and serving as a foundation for evidence-based policymaking and programming. This systematic literature review aimed to address this gap by analysing research on the fostering and assessment of 21st century skills in sub-Saharan Africa. By mapping existing studies and interventions, this review sought to provide insights into effective strategies, highlight contextual barriers, and inform future policy and practice.

The following research questions guided the systematic review:

1. What approaches are currently used to nurture 21st century life skills among young people in sub-Saharan Africa?
2. What promising interventions and best practices can be identified from the existing literature?
3. Which tools and methods are employed to assess these skills in the region, and how effective are they?

## 3 Methodology

**Figure 1:**
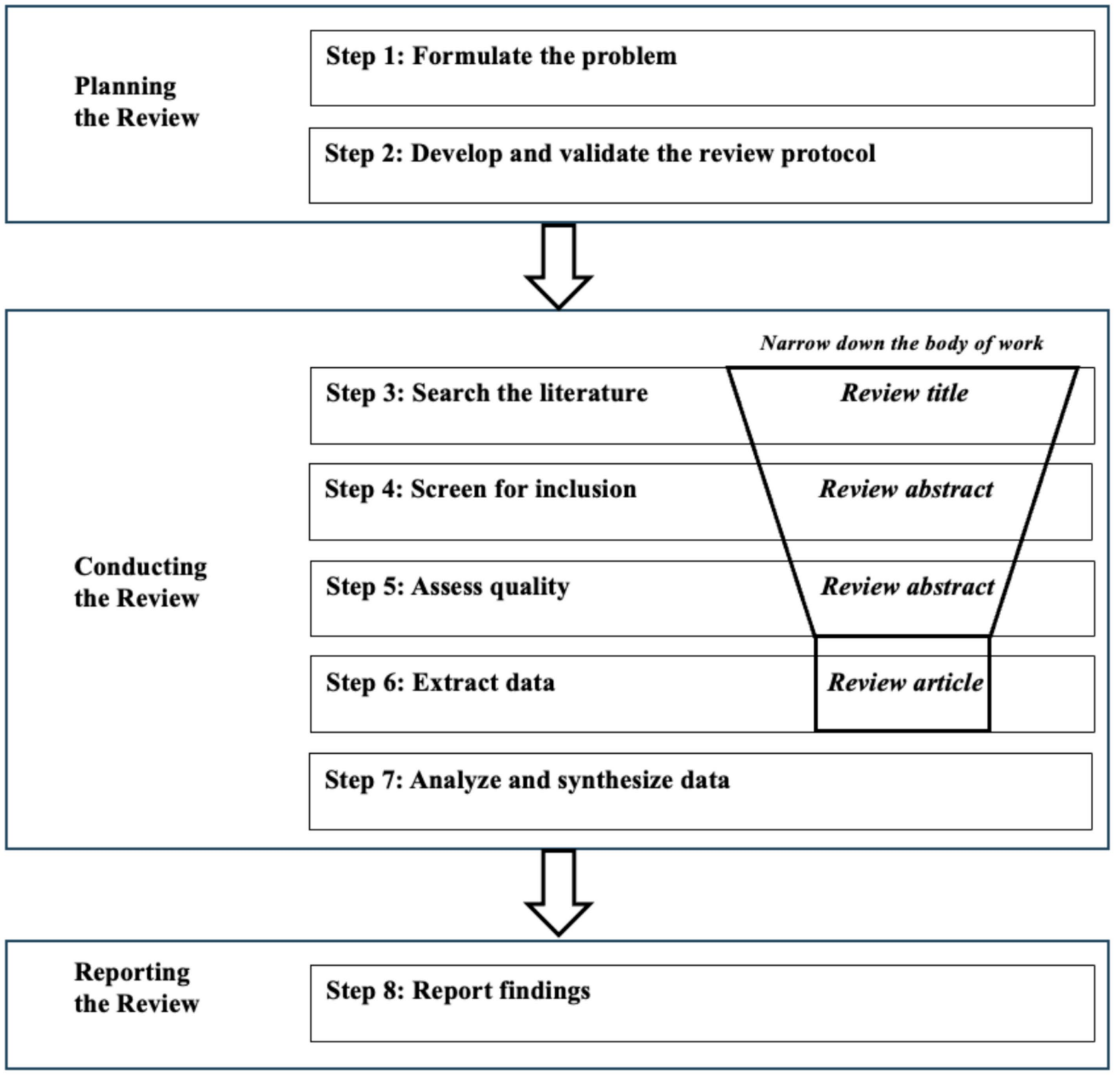
Summary of protocol for the systematic review.

We conducted a systematic review to understand the landscape of nurturing approaches and assessment of life skills in sub-Saharan Africa. We developed a systematic review protocol, summarised in Figure 1, describing the plan for the review, the research purpose, the search strategy, inclusion and exclusion criteria, data collection and analysis strategy and reporting structure. We conducted a search for academic and grey literature.

### 3.1 Data search and extraction strategy

The search strategy followed a systematic review methodology as codified by the Cochrane Collaboration. Firstly, we conducted a systematic search for relevant literature in both academic and organisational databases (see Table 1).

**Table 1:** Databases searched.

Academic databases searched	Organisational databases searched
EBSCO Host; ERIC; ProQuest Central; PsychInfo; Science Direct; Scopus; SocINDEX	Abdul Latif Jameel Poverty Action Lab (J-PAL); World Bank eLibrary; Inter-Agency Network for Education in Emergencies (INEE); International Initiative for Impact Evaluation (3ie); International Development Research Center (IDRC); Overseas Development Institute (ODI); ODI Humanitarian Practice Network (HPN)

We delimited our searches to the years 2018-present (November, 2023) and used three categories: (i) location, (ii) age group, (iii) 21st century skills, and (iv) intervention or assessment. For location (i), we included all countries in sub-Saharan Africa and “Africa.” For age groups (ii), we included children, youth, and synonyms for formal and nonformal education. We also included synonyms and related fields for “21st century skills” (iii), including, but not limited to, life skills, 21st-century skills, social–emotional learning, and psychosocial support. Finally, we included the descriptors “intervention” or “assessment” to capture records reporting elements of life skills interventions or assessments (see Table 2).

**Table 2:** Search terms.

Categories	Descriptors
Location	“Africa” or “Sub-Saharan” or “subsaharan” or “sub saharan” or “Zanzibar” or “Angola” or “Benin” or “Botswana” or “Burkina Faso” or “Burundi” or “Cabo Verde” or “Cameroon” or “Central African Republic” or “Chad” or “Comoros” or “Congo” or “Cote d’Ivoire” or “Equatorial Guinea” or “Eritrea” or “Eswatini” or “Swaziland” or “Ethiopia” or “Gabon” or “Gambia, The” or “Ghana” or “Guinea” or “Guinea-Bissau” or “Kenya” or “Lesotho” or “Liberia” or “Madagascar” or “Malawi” or “Mali” or “Mauritania” or “Mauritius” or “Mozambique” or “Namibia” or “Niger” or “Nigeria” or “Rwanda” or “Sao Tome and Principe” or “Senegal” or “Seychelles” or “Sierra Leone” or “Somalia” or “South Africa” or “South Sudan” or “Sudan” or “Tanzania” or “Togo” or “Uganda” or “Zambia” or “Zimbabwe”
Lifeskills synonyms	“21st century “or “twenty first century” or “twenty-first century” or “non cognitive” or “non-cognitive” or “positive youth development” or “psycho-social” or “psycho social” or “psychosocial” or “SEL” or “social and emotional” or “socioemotional” or “social–emotional” or “social emotional” or “socio-emotional” or “soft skills” or “transferrable skills” or “whole child” or “employability skills” or “character skills” or “life skills” or “lifeskills” or “life-skills” or “transversal skills”
Age group	“children” or “basic education” or “primary school” or “primary education” or “secondary education” or “secondary school” or “extracurricular” or “after-school” or “after school” or “classroom” or “informal education” or “non-formal education” or “nonformal education”
Intervention or assessment	“intervention” or “assessment”

The academic databases enabled the use of all categories and descriptors concurrently in the search strings, allowing for a comprehensive and systematic retrieval of records. By contrast, the organisational databases did not always permit the simultaneous application of multiple descriptors. In these cases, searches had to be conducted progressively, combining terms to approximate the same level of coverage.

Our searches initially returned more than 4,000 publications. After removing duplicates, reviewers screened the abstracts and excluded records not referring to an intervention or assessment on life skills or not related to the geographical scope of interest. This process resulted in 167 publications being retained for full-text screening, comprising 148 from academic databases and 19 from organisational databases.

Secondly, we retrieved the full reports and reviewed them against the inclusion criteria. From the academic databases, we excluded 49 reports because the beneficiaries were not within the 6–17 age range, 9 because they were not focused on life skills, 1 because it was outside the geographical scope, and 60 because they did not assess an intervention on life skills nurturing. From the organizational databases, we excluded two reports because the beneficiaries were not within the 6–17 age range, 1 because it was not focused on life skills, 1 because it was outside the geographical scope, and 8 because they did not assess an intervention on life skills nurturing. The remaining report was a duplicate of one already retrieved from the academic databases and was therefore excluded.

**Figure 2:**
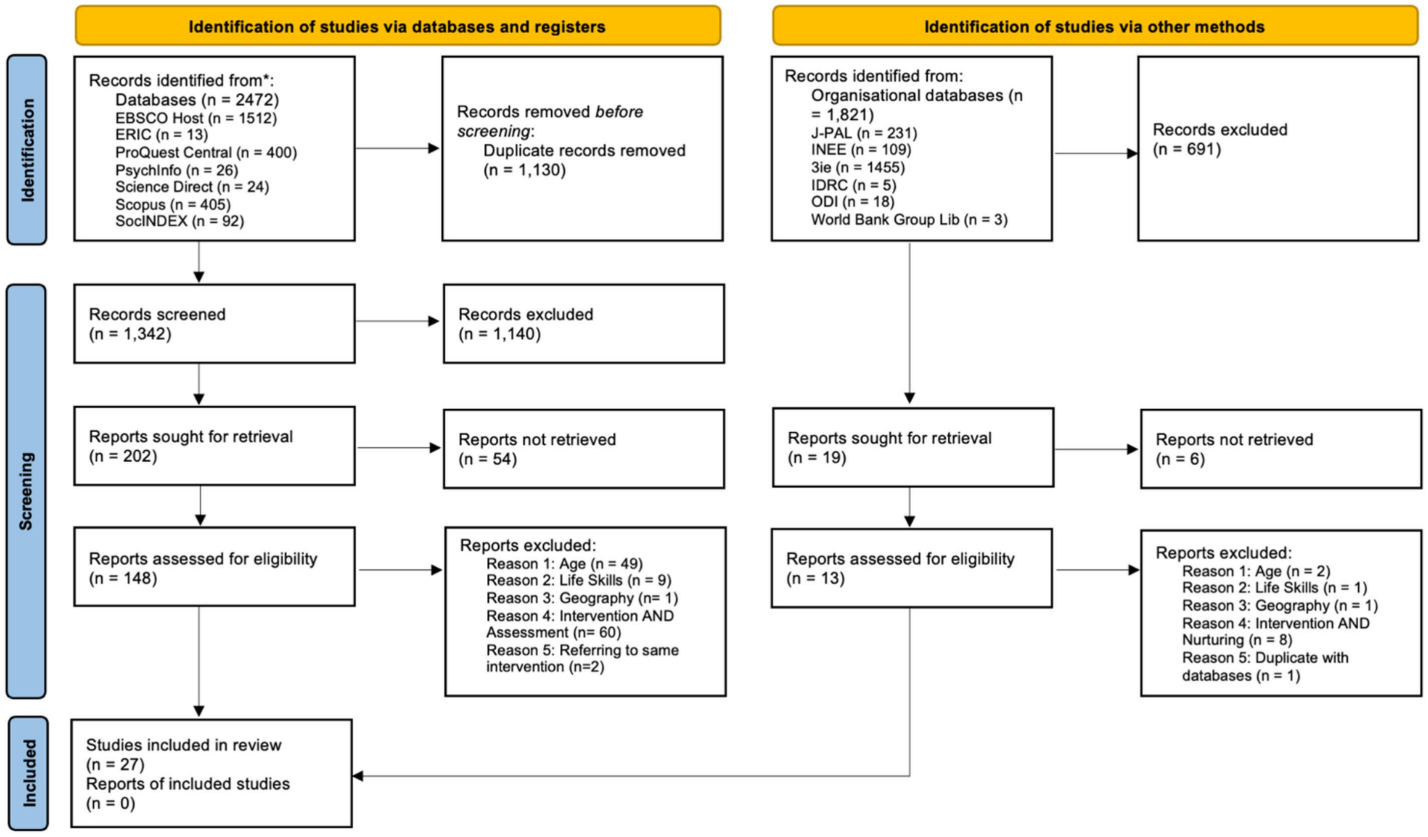
Systematic literature review extraction strategy (PRISMA 2020 flow diagram). Source: Page et al. (2021).

We therefore conducted a third full review of the 29 publications and found that two interventions were reported in duplicate. As a result, only 27 unique interventions were included in the analysis (see Figure 2).

### 3.2 Data extraction and quality assessment

A structured data extraction form in Excel was developed for this review, adapted to the specific focus on 21st century life skills. The form captured key study characteristics (author, year, country, study design, and population), intervention details (setting, target group, skill domains addressed, duration, and delivery mode), assessment tools employed (type of instrument, domains measured, validity, and reliability where reported), and outcomes (life skills, psychosocial, educational, and livelihood-related).

Data extraction was undertaken by one primary reviewer for each database, while a second reviewer independently verified the extracted data against the original publication. This two-step process was applied to ensure accuracy, reduce individual bias, and improve reliability. Disagreements between reviewers were resolved through discussion and consensus, with recourse to a third senior reviewer where necessary.

Consistency in data extraction was further ensured by piloting the extraction form on a subset of five studies and refining the categories and definitions prior to completing the process for all included studies. This calibration step helped align interpretations across reviewers and ensured comparability of extracted data. In addition, a screenshot of the extraction page for each database search was saved and shared in a common folder accessible to all reviewers. This served as an additional quality control measure, allowing for cross-checking of search results and verification of extracted data.

### 3.3 Data analysis and quality assessment

We extracted data on the intervention, including the targeted domains, intervention approach, and facilitator profile. We also summarised the intervention’s impact when available. We collated the specific assessment tool used, the effect sizes (where applicable), the impact (i.e., positive, negative, null, or mixed), the intervention aims, and the approach to training on life skills. We focused on the assessment tools used, including contextualisation and validation processes and challenges the researchers faced.

The appraisal of the quality of included studies considered aspects such as clarity of research objectives, appropriateness of methodology, adequacy of sample size, transparency in reporting, and robustness of outcome measures. Each study was independently assessed by two reviewers, with disagreements resolved through consensus. Quality ratings were not used as an exclusion criterion; rather, they informed the interpretation of findings and the weight given to evidence in the synthesis. Studies of lower methodological quality were included in the review to ensure breadth of evidence but are discussed with caution in the results and discussion sections.

### 3.4 Study selection process

The study selection process was conducted systematically to ensure rigour and consistency. For each database search, a primary searcher executed the search strategy and documented all search terms, Boolean operators, and filters applied. A secondary reviewer independently checked the search outputs for completeness and relevance. Title and abstract screening were carried out independently by two reviewers, with discrepancies discussed and resolved through consensus. In cases where agreement could not be reached, a third reviewer adjudicated the decision.

The decision to limit the searches to January 2018 onwards was guided by the rapid evolution of 21st century skills frameworks and assessment methodologies in sub-Saharan Africa in recent years, particularly in the wake of increased policy attention and programmatic activity following the Sustainable Development Goals (SDGs) agenda. This timeframe ensured that the review focused on contemporary evidence and methodologies that reflect current educational priorities, technological contexts, and policy frameworks.

## 4 Findings

The findings are represented in two broad categories: approaches to nurturing life skills in sub-Saharan Africa and assessment tools used in measuring life skills in sub-Saharan Africa, respectively. Each category outlines a detailed summary of findings, followed by a synthesis of emerging themes and discussions of key implications.

### 4.1 Findings on nurturing approaches for life skills in sub-Saharan Africa

To answer the first research question on what approaches are currently used to nurture 21st century life skills among young people in sub-Saharan Africa, the selected reports were analysed. The interventions in the 27 articles reviewed span both school-based and community-based settings, addressing diverse approaches to developing life skills and improving child and adolescent well-being. Thirteen articles focused on school-based interventions, and 14 on community-based interventions. Each of the two categories is described in more detail below.

#### 4.1.1 Description of school-based interventions

School-based interventions include in-classroom interventions as well as extracurricular interventions. Five (5) were classroom-focused interventions and investigated the impact of in-classroom activities (Berger et al., 2018; Kibga et al., 2021; McMullen and McMullen, 2018; Sebatana and Dudu, 2022; Torrente et al., 2019), while eight (8) articles explored activities beyond the traditional classroom environment (Ambelu et al., 2019; Cherewick et al., 2021; Merrill et al., 2018; Millanzi et al., 2022; Ashraf et al., 2020; Ndetei et al., 2019; Olowokere and Okanlawon, 2018; Pleaner et al., 2022).

The five classroom-based interventions targeted adolescents aged 10–18. Two of these interventions focused on skilling interventions tailored to specific subjects (Kibga et al., 2021; Sebatana and Dudu, 2022) while developing specific skills. Kibga et al. (2021) for instance, focused on developing learners’ curiosity under a teachers’ guidance, while Sebatana and Dudu (2022) explored how Grade 10 teachers enhanced communication and critical thinking through problem-based learning (PBL). Berger et al. (2018) emphasised resiliency skills through practices such as perspective-taking, empathy training, mindfulness, and compassion cultivation.

The content of the five classroom-based interventions aimed at enhancing students’ skills and wellbeing. Examples of promising interventions include incorporating hands-on activities and using locally-sourced materials to increase students’ curiosity within chemistry classes in Tanzania (Kibga, et al., 2021), employing problem-based learning (PBL) and pedagogy in science education in South Africa (Sebatana and Dudu, 2022), utilising problem-based learning to enhance critical thinking, creativity, communication, and problem-solving skills integrating reproductive health content into Biology and Civics in Tanzania (Millanzi et al., 2022). Another program, the Learning in a Healing Classroom (LHC) intervention, included materials and strategies within lessons to create emotionally supportive and cooperative learning environments (Torrente et al., 2019).

Extracurricular interventions refer to the school-based interventions outside the classroom. These targeted general school populations of various age groups, ranging from 10 to 18 years. The interventions mainly aimed at improving life skills acquisition (Ndetei et al., 2019), health knowledge (Merrill et al., 2018; Millanzi et al., 2022), interpersonal skills (Ashraf et al., 2020), supporting the development of social emotional mindsets and skills (Cherewick et al., 2021) and improve psychosocial wellbeing (Ambelu et al., 2019). Key skills fostered by the studies include self-awareness, empathy, stress and emotion coping, communication, relationship building, problem solving, critical thinking, decision making, and interpersonal skills.

These programs provided training on safety (Stark et al., 2018a,b; Austrian et al., 2021), health and livelihoods (Packer et al., 2020), health and financial skills (Austrian et al., 2020), specific life skills that were deemed necessary for girls (negotiation skills in Ashraf et al., 2020 and self-esteem/efficacy in Merrill et al., 2018). All programs targeting adolescent girls also included sexual and reproductive health (SRH) knowledge and/or skills training as a component or the primary focus of the program (Pleaner et al., 2022). Two studies specifically targeted creating safe spaces for refugee adolescent girls (Stark et al., 2018a,b). One study highlighted the role of technology in facilitating social, emotional, and identity learning, emphasising skills such as growth mindset, curiosity, generosity, persistence, purpose, and teamwork (Cherewick et al., 2021). One intervention targeted building positive coping strategies and resilience among children in high-stress environments (Ambelu et al., 2019). Olowokere and Okanlawon (2018) focused on six core characteristics of resilience—equanimity, meaning, perseverance, self-esteem, self-reliance, and existential aloneness, foster self-development as well as foster a supportive and resilient community for children to thrive.

Several methodologies were adopted including (i) use of animated modules targeting growth mindset, curiosity, generosity, persistence, purpose, and teamwork, engaging children with storylines and songs designed to develop these key social and emotional skills (Cherewick et al., 2021); (ii) use of games and sports with structured discussions and activities on sexual reproductive health, and HIV knowledge to build decision making skills and link participants with health services (Merrill et al., 2018); (iii) use of problem-based pedagogy to enhance decision making skills and safe behaviours (Millanzi et al., 2022); (iv) curated stand-alone curriculum targeting specific need based skills development (Ndetei et al., 2019; Ashraf et al., 2020; Olowokere and Okanlawon, 2018); and (v) multifaceted approach which combines a number of approaches into one (Pleaner et al., 2022; Merrill et al., 2018).

#### 4.1.2 Description of community based interventions

Community-based interventions, detailed in 14 articles, concentrated on creating supportive environments outside the formal school setting. The selected studies implemented diverse interventions targeting children aged 6–18 across various countries, aimed at improving different facets of children’s lives, from social and economic empowerment to psychological well-being, resilience, and protection against violence.

A significant cluster of interventions focused on enhancing social as well as economic assets. For instance, Austrian et al. (2020) addressed social and economic assets by boosting self-efficacy, creating safe spaces for girls to meet friends, promoting positive gender attitudes, and fostering non-acceptance of intimate partner violence.

Interventions focusing on psychological wellbeing and resilience formed another distinct cluster. Harding et al. (2019) utilised an ecological model to enhance children’s resilience by building on their strengths, peer relationships, and community support. Hermosilla et al. (2019) focused on psychosocial wellbeing. Hosaka et al. (2021) monitored resilience improvements among peer leaders. Ismayilova et al. (2018) focused on mental health by measuring depression, self-esteem, and trauma symptoms. Kachingwe et al. (2021) aimed at improving early child development and the psychosocial wellbeing of adolescent mothers by focusing on self-esteem and resilience. Metzler et al. (2021, 2023) focused on reducing psychological distress and enhancing resilience among children.

A third cluster of interventions targeted protection and health. Stark et al. (2018a) worked on improving communication skills, supporting adolescent girls, and understanding violence and abuse, while a subsequent study by Stark et al. (2018b) aimed at reducing exposure to sexual and physical violence, neglect, child marriage, and transactional sex. Willis et al. (2019) evaluated various aspects of psychological well-being, including self-esteem and stigma, as well as adherence to treatment and retention in care for adolescents living with HIV.

Through structured activities, mentorship, and community engagement, these programs aimed at empowering children and adolescents, promoting wellbeing and personal development. Harding et al. (2019) conducted a five-day residential camp offering activities like Memory box, Memory book, Tree of life, and the Hero book, facilitated by qualified social workers, a clinical officer, and a medical doctor. Metzler et al. (2023) delivered locally adapted psychosocial programming through structured and unstructured activities over 12 weeks. Austrian et al. (2020) emphasised the significance of weekly girls’ group meetings, facilitated by female mentors, as a core component for improving health and economic outcomes. These safe spaces provided opportunities for discussions and empowerment sessions. Kachingwe et al. (2021) established safe and inclusive community spaces for adolescent mothers and pregnant girls, hosting dialogue sessions twice a month to address their needs and concerns. Nwokedi et al. (2023) utilised Interactive Radio Instruction (IRI) and Interactive Television (ITV) to teach life skills to nomadic children. Facilitators assisted during radio and TV classes, ensuring a smooth learning experience and interaction with the instructional content.

#### 4.1.3 Impact of interventions nurturing life skills

To answer the second research question on what promising interventions and best practices can be identified from the existing literature, we analysed the results reported by the 27 articles. The interventions supporting the nurturing of life skills both in and out of the classroom demonstrated positive results. Almost all studies (*n* = 20) reported positive effects on at least one outcome of interest, as can be seen from Table 3.

**Table 3:** Overall impact of interventions nurturing 21st century skills.

Impact direction	# of studies
Positive	20
Negative	0
Null	2
Mixed	4
Unknown	1

##### 4.1.3.1 Targeted and structured, experiential and active instruction works

The majority of studies reported findings from a structured, sequenced, and lesson-plan-based intervention over a period of months or years. These all led to some positive gains in the outcomes measured, while the less structured mentorship groups reported limited and mixed effects. These structured curricula cite active, experiential, and problem-based learning as essential to their approaches. A number of studies specifically highlighted the experiential and active nature of their pedagogical approach (Berger et al., 2018; Kibga et al., 2021; Merrill et al., 2018; Metzler et al., 2023; Millanzi et al., 2022; Olowokere and Okanlawon, 2018), though it is likely that other programs also used experiential pedagogies. Although the literature is scant on the comparative effects of different approaches on the same, or even related, measures, we found two studies that compared more and less structured intervention approaches and two studies that tested different pedagogical approaches. Based on these empirical comparisons, targeted and experiential approaches to teaching life skills demonstrated larger gains in life skills and related outcomes. Both studies found that participants in the more targeted approach saw overall greater gains (Metzler et al., 2023; Olowokere and Okanlawon, 2018).

In Uganda, Metzler et al. (2023) compared a “toolkit” intervention, which provided an interactive session with 40 sequenced lessons, to the “standard” child-friendly space intervention that included structured and unstructured recreational activities. Although both interventions and a control group reported improvements in psychosocial distress, resilience, and developmental assets, children in the two interventions had overall greater outcomes than the control. Moreover, the toolkit led to greater gains on some outcomes in comparison to the standard or control. The more structured toolkit led to greater gains on relational and overall resilience scores than the less structured standard approach (*b* = 0.927, *p* = 0.025; and *b* = 1.881, *p* = 0.033) and statistically significantly greater gains in developmental assets than the control group (*b* = 1.908, *p* = 0.013). Similarly, in Nigeria, Olowokere and Okanlawon (2018) compared a structured resilience-based training curriculum to a peer support group over a period of 6 weeks. Trained teachers taught the resilience group using participatory learning approaches, and although the peer group was facilitated by teachers, it was led by the students and intended to be less structured. The resilience group reported significantly lower anxiety scores than the peer group at three (Md = −2.93, *t*(337) = −3.17, *p* = 0.002) and six (Md = 2.62, *t*(334) = −2.76, *p* = 0.006) months and significantly higher self-esteem scores at 3 months (Md = 0.85, *t*(337) = 2.19, *p* = 0.029). In both studies, the researchers recommend prioritising the more structured “toolkit” and “resilience” approaches, respectively.

Two studies compared three arms of problem-based and experiential learning with more traditional, lecture-based pedagogical approaches. In one study, Cherewick et al. (2021) tested three variations of the Discover Learning social emotional learning program in Tanzania. The A group only had content learning, the B group added reflection, and the C group also added experiential practice. Group C which had experiential practice and reflection, saw greater gains in all outcomes in comparison to groups B and A, including an effect size using Cohen’s d of 0.45 compared to 0.15 (B) and 0.33 (A) on self-efficacy; 1.09 compared to 0.15 (B) and 0.93 (A) on Growth Mindset. Another study tested different pedagogical approaches to integrating reproductive health lesson materials in biology and civics clubs in Tanzania. The researchers compared pure problem-based pedagogy (PBP) with a hybrid PBP and a lecture-based pedagogy (LBP). Students in both the pure and hybrid PBP had higher soft skills than LBP, but there was no significant difference between the two forms of PBP. Specifically, the level of soft skills measured with a beta coefficient was significantly higher in the hybrid PBP (*β* = 9.0986, *p* < 0.01; 95%CI: 4.7772, 14.2311) and pure PBP (*β* = 8.7114, *p* < 0.01; 95%CI: 3.9990, 10.1208) than in the control group (Millanzi et al., 2022). In both studies, a problem- and experiential-based pedagogical approach was more effective in improving life skills than a lecture or content-focused approach.

##### 4.1.3.2 Focus on the internal domain before the external

Life skills programs can target internal, or “self”-focused domains and/or external, or “social”-focused domains. More studies focused on the “self” domain first, whether or not they also included social skills. We found eight studies that measured self-esteem and self-efficacy as a primary outcome. Five employed the Rosenberg self-esteem scale (Ismayilova et al., 2018; Merrill et al., 2018; Olowokere and Okanlawon, 2018; Harding et al., 2019; Kachingwe et al., 2021), and four used self-efficacy scales—the general self-efficacy scale (McMullen and McMullen, 2018; Austrian et al., 2020) or the Self-Efficacy Questionnaire for Children (SEQC) (Harding et al., 2019; Cherewick et al., 2021). Other studies also measured other individual-level competencies, such as resilience, anxiety, and hope. Below, we describe two programs that saw positive effects and focused on both the self and prosocial competencies, followed by qualitative data from two studies.

The Living Well program in Uganda trained secondary school students on life skills within the classroom. The content was adapted from other life skills programs in Uganda, Kenya, and the DRC and tailored to the specific context. It targeted both individual and prosocial skills across four themes: (1) Living Well with Ourselves and Others; (2) Living Well with Worry and Stress; (3) Living Well with Life’s Issues; and (4) Living Well in the Future. Compared to a control group, the intervention group had significant positive effects in general self-efficacy [*F*(1,167) = 19.66, *p* < 0.001, η^2^ = 0.106], internalising problems [*F*(1,167) = 10.58, *p* = 0.001, η^2^ = 0.060], and overall “Connectedness” [*F*(1,167) = 15.24, *p* < 0.001, η^2^ = 0.085], and a small, but statistically not significant increase in prosocial attitudes [*F*(1,167) = 5.61, *p* = 0.019, η^2^ = 0.033]. The gains in “connectedness” appear to be primarily due to changes in connectedness to self-in-the-present [*F*(1,167) = 12.87, *p* < 0.001, η^2^ = 0.072] and self-in-the-future [*F*(1,167) = 9.81, *p* < 0.002, η^2^ = 0.056] (McMullen and McMullen, 2018). A good example of this trend of focusing on the self-domain first is the “ERSAE-Stress-Prosocial (ESPS)” intervention, even though the researchers did not assess self-efficacy or self-esteem. The primary assessment tool was the Strengths and Difficulties Questionnaire (SDQ). ESPS provided a culturally adapted, universal school-based, structured and manualised intervention in Tanzania that targeted both individual-level and prosocial skills. The program begins by addressing stress-related reactions and teaching coping strategies, then aims at building prosocial skills, such as empathy. This scaffolded approach demonstrated positive impacts post-intervention and 8 months follow-up (Berger et al., 2018).

Moreover, qualitative data suggested that “self-focused” skills were the foundation for building other skills and positive outcomes. In Mozambique, the Women First intervention used the Go Girls! Life skills curriculum alongside an economic empowerment program. The qualitative study found that “having hope and self-efficacy and behavioural competence enabled progress towards achieving goals […] fostering individual-level protective factors (e.g., hope and self-efficacy) is useful in interventions targeted towards young women to prevent pregnancy and child marriage, but that external assets (e.g., community, structural interventions) should be promoted to facilitate girls staying in school, [etc.]” (Packer et al., 2020, p. 580). The researchers found that both hope and self-efficacy are necessary components of goal achievement. Those girls who were both hopeful and had self-efficacy were on track to reach goals, while those who were only hopeful with mixed levels of self-efficacy were not on track to reach goals, and those that lacked both hope and self-efficacy were not taking any actions to reach their goals.

Similarly, the mixed-methods assessment of the SKILLZ Street soccer program found that the self-focused skills they gained (Rosenberg self-esteem: ES = 0.25) enabled them to build other, internal and external skills. “SKILLZ Street gives you the confidence, like strong body language. Not only will I use it to say no to sex, but for other things as well. If you do not want it, “No.” (SKILLZ Street Participant). [The soccer coaches] suggested that the program builds participants’ self-esteem, enabling them to discover themselves while recognising their strengths and weaknesses” (Merrill et al., 2018, p. 17). Based on the mixed-methods findings across these studies, the focus on self-skills followed by prosocial skills seems promising.

### 4.2 Findings on assessment of life skills in sub-Saharan Africa

Beyond the approaches adopted to nurture life skills, an equally important dimension concerns the ways in which these skills are assessed (see Research Question 3). Understanding which tools and methods have been employed in sub-Saharan Africa, and evaluating their effectiveness, provides insight into both the strengths and the limitations of current practices. The following section addresses this question by examining the assessment instruments reported in the reviewed studies and discussing their adequacy in capturing the multidimensional and contextual nature of life skills.

#### 4.2.1 Tools used in measuring 21st century skills in sub-Saharan Africa

The study findings provide a detailed overview of tools used in measuring 21st century skills in sub-Saharan Africa. The tools and measurements targeted early childhood development (3–6), children (6–12 years old) and adolescents and youth (13–19 years old). Findings revealed that forty-eight (48) different tools were used to measure a range of skills and attributes across cognitive and academic domains (e.g., literacy, numeracy, problem solving, critical thinking, and goal orientation); psychosocial and emotional domains (resilience, decision making, self-esteem, empathy, social connectedness, communication, interpersonal relationships, and coping strategies); and mental health and wellbeing (emotional and behavioural problems, anxiety, depression, and psychological wellbeing).

The majority of the measures in these studies targeted skills broadly categorised to strengthen psychosocial wellbeing (*n* = 16 studies), self-esteem or self-efficacy (*n* = 8 studies), social assets (*n* = 6 studies), or resilience (*n* = 4 studies). Measures that included a composite for multiple domains were primarily developed outside of sub-Saharan Africa. They either measured psychosocial wellbeing, including the Strengths and Difficulties Questionnaire (*n* = 4) and the African Youth Psychosocial Assessment (*n* = 1), or social and developmental assets, including the Developmental Assets Protocol (*n* = 3), the Hemingway Measure of Adolescent Connectedness (*n* = 1) or measures created by the researcher. There is a clear gap in the assessment tools used to assess 21st century skills in sub-Saharan Africa.

#### 4.2.2 Approaches used in assessing 21st century skills in sub-Saharan Africa

The systematic review highlighted that most tools used in measuring 21st century skills in sub-Saharan Africa were questionnaires and self-report measures. Examples include the Rosenberg Self-Esteem Scale (Harding et al., 2019; Kachingwe et al., 2021; Olowokere and Okanlawon, 2018), the Connor Davidson Resilience Scale (CD-RISC) (Hosaka et al., 2021), the Child and Youth Resilience Measure (CYRM) (Ambelu et al., 2019), the Youth Self Report (Ndetei et al., 2019), the African Youth Psychosocial Assessment Instrument (AYPA) (McMullen and McMullen, 2018), the Strengths and Difficulties Questionnaire (SDQ) (Harding et al., 2019; Hermosilla et al., 2019; Torrente et al., 2019); the Hemingway Measure of Adolescent Connectedness (McMullen and McMullen, 2018); Relationship With Teacher Questionnaire (Torrente et al., 2019), among others. While these assessments can be administered quickly and at a low cost, they rely heavily on the honesty and self-awareness of the respondents, which can sometimes lead to biassed results.

#### 4.2.3 Tool contextualisation and validation processes

The study revealed a glaring gap in the literature on context-driven tools for measuring 21st century competencies. The contextualisation processes emerged in the review were mainly translation and adaptations. Many tools underwent backward and forward translation to ensure linguistic and conceptual equivalence. For instance, the Rosenberg Self-Esteem Scale (Harding et al., 2019; Merrill et al., 2018) and the Brief Resilience Scale (Kachingwe et al., 2021) were translated and adapted to local languages and contexts. In a number of cases, tools were piloted to assess their reliability and validity in the new context (Kibga et al., 2021; Olowokere and Okanlawon, 2018; Torrente et al., 2019). In some cases, local experts and facilitators were involved in the adaptation process to ensure that tools are culturally appropriate and contextually relevant (Ndetei et al., 2019; McMullen and McMullen, 2018; Metzler et al., 2023). In other contexts, tools were adapted through interactive group discussions with local stakeholders, followed by pilot testing with target populations (Ndetei et al., 2019; Kibga et al., 2021; Olowokere and Okanlawon, 2018). The tool validation processes relied heavily on confirmatory factor analysis, reliability testing, and content validity testing. In some cases, expert panels and focus groups validate the content and ensure that it accurately reflects the measured constructs (Millanzi et al., 2022; Willis et al., 2019). The contextualisation process highlights the importance of ensuring that assessment tools are contextually appropriate and culturally sensitive, enhancing their effectiveness in measuring competencies.

## 5 Discussion

The evidence reviewed confirms a consistent pattern: structured, sequenced, and experiential approaches—whether in school or community settings—are more effective at nurturing life skills than unstructured or purely didactic interventions. This aligns with broader global literature on life skills education, which emphasises active, participatory, and learner-centred pedagogies (UNESCO, 2017). The emphasis on internal or “self-focused” skills (e.g., self-esteem, self-efficacy) as a precursor to social and prosocial skills echoes socio-emotional learning frameworks (CASEL, 2017) and ecological models of child development (Bronfenbrenner, 1992), where personal competencies provide the foundation for interpersonal and societal engagement. The review also reinforces global concerns regarding measurement: while there is an increasing adoption of tools such as the Rosenberg Self-Esteem Scale, Strengths and Difficulties Questionnaire, and Child and Youth Resilience Measure, the majority of these were developed outside sub-Saharan Africa and require contextual adaptation. This is consistent with calls in the literature for region-specific, culturally grounded assessment instruments (Jukes and Norman, 2024). In line with international trends, the evidence suggests that combining life skills training with broader protective, health, and economic empowerment interventions (Packer et al., 2020; Austrian et al., 2021) generates more holistic and sustainable outcomes, particularly for adolescent girls and other vulnerable groups.

The findings of this review have several important implications for practice, policy, and research. From a programmatic perspective, the evidence underscores the value of structured, experiential, and contextually relevant pedagogies in fostering life skills. Interventions that scaffold self-focused skills—such as self-awareness, self-esteem, and self-efficacy—before progressing to prosocial competencies tend to be more effective, suggesting the importance of a developmental sequencing in program design. Embedding life skills within both formal school curricula and community-based platforms is essential for ensuring broad coverage, particularly for out-of-school children and adolescents who might otherwise be excluded from such opportunities. Moreover, multi-component approaches that integrate life skills education with economic empowerment, health services, and the provision of safe spaces appear particularly beneficial for adolescent girls, enhancing both immediate outcomes and long-term resilience.

At the policy level, there is a clear case for recognising life skills as core competencies within national education frameworks. This requires not only formal inclusion in curricula but also the provision of targeted teacher training in active, learner-centred pedagogies that move beyond rote instruction. Policy actors, including ministries and development partners, should also prioritise the development and validation of culturally appropriate assessment tools, enabling more accurate and context-sensitive measurement of outcomes. Furthermore, multi-sectoral collaboration—linking education, health, and social protection—can help ensure that interventions are both comprehensive and sustainable, addressing the interlinked determinants of young people’s development.

Future research should address critical gaps identified in the current evidence base. Longitudinal studies are particularly needed to understand the durability of life skills gains over time and to capture delayed or cumulative impacts. Comparative effectiveness trials could help identify the optimal combinations of delivery modality, duration, and content for different population groups, thereby informing both efficiency and scalability. Engaging youth directly in the design of assessment tools and intervention strategies will be key to ensuring that such efforts are meaningful and relevant to their lived realities. Finally, systematic evaluations of implementation fidelity and scalability—especially in low-resource and crisis-affected contexts—will be vital for translating promising models into sustainable, large-scale practice.

### 5.1 Limitations

Although the review highlights promising results, several limitations within the evidence base warrant careful consideration.

One persistent concern is the likelihood of publication bias. The majority of included studies reported positive findings, with few documenting null or negative results. This imbalance may overstate the apparent effectiveness of life skills interventions and obscure important lessons from less successful or inconclusive programs.

Another limitation arises from the considerable heterogeneity in intervention design. Differences in target populations, program duration, delivery modalities, and the specific life skill domains addressed make direct comparisons difficult and limit the generalisability of findings. Such variability also complicates efforts to synthesise results across studies, as the same label of “life skills” may encompass markedly different content and pedagogical approaches. The temporal scope of most evaluations further constrains the evidence. With follow-up periods typically lasting only a few months, it remains uncertain whether observed gains in life skills are sustained over the long term, particularly in the face of changing socio-economic or educational conditions.

Measurement practices also present notable challenges. Many studies rely heavily on self-reported data, which can be influenced by social desirability bias—an especially relevant concern when assessing sensitive topics such as sexual and reproductive health or psychosocial well-being. The absence of triangulation with objective or observational measures reduces confidence in the robustness of reported outcomes. Cost-effectiveness data are strikingly limited. Without systematic economic evaluation, it is difficult to determine which interventions offer the best return on investment, a critical consideration for scaling programs in resource-constrained settings. Collectively, these limitations highlight the need for more rigorous, longitudinal, and contextually grounded research to inform both policy and practice.

In addition, this review was not prospectively registered in a systematic review registry. At the time of conducting the review, protocol registration was not a compulsory requirement in our institutional or national context (Uganda). Nonetheless, we acknowledge that prospective registration is considered best practice for systematic reviews, as it promotes transparency, reduces the risk of bias, and facilitates reproducibility. The absence of protocol registration is therefore a limitation of this work, and future reviews should seek to align with international standards by registering protocols prior to commencement.

## 6 Conclusion

This systematic review set out to address a clear research gap: while the development of 21st century skills is widely recognised as essential, evidence from sub-Saharan Africa remains fragmented, inconsistent, and often disconnected from locally relevant assessment practices. Our analysis confirms that the existing literature is both promising and constrained by significant methodological and contextual challenges.

Although many studies reported positive outcomes from life skills interventions, few measured general life skills directly. More often, life skills programming served as a vehicle to improve other domains—such as health, safety, education, or economic resilience—without systematically assessing the targeted competencies themselves. This reflects a broader issue in the region: the absence of common, context-appropriate frameworks and tools for measuring 21st century skills. Without these, it is difficult to determine which approaches are most effective, for whom, and under what conditions.

Despite this fragmentation, several insights emerge that can guide the field towards more coherent and impactful practice. Effective interventions tend to be structured, targeted, and sequential—scaffolding self-focused skills (such as self-awareness and self-efficacy) before building prosocial competencies (such as collaboration and empathy). These approaches are most impactful when grounded in experiential and active learning that connects to learners’ lived realities, facilitated by trained educators or community actors, and delivered within safe, supportive environments. Teacher professional development, stakeholder engagement, and alignment with national curricula were recurrent features of successful models, particularly when adapted for vulnerable groups such as out-of-school adolescents, refugees, and young people living with HIV.

A critical barrier to progress is the lack of robust, culturally validated assessment tools. Many programs rely on instruments developed in non-African contexts or on tools not designed to capture life skills development over time. This not only hampers comparability but also limits the capacity of policymakers and practitioners to track progress, evaluate cost-effectiveness, and make evidence-based decisions.

Addressing these gaps requires a coordinated research agenda that combines rigorous evaluation designs, longitudinal tracking, and participatory approaches to tool development. Cross-country collaboration could enable the creation and validation of regionally relevant assessment frameworks, while comparative effectiveness studies could help identify scalable models suited to diverse contexts.

By mapping the current evidence, this review provides a foundation for moving from fragmented, isolated interventions towards coherent, evidence-based strategies for nurturing and assessing 21st century skills in sub-Saharan Africa. Closing the research–practice gap will require sustained investment in teacher capacity, context-sensitive measurement, and multi-sectoral partnerships—ensuring that life skills development is not only a policy aspiration but a measurable, sustainable reality for all young people in the region.

## Data Availability

The original contributions presented in the study are included in the article/Supplementary material, further inquiries can be directed to the corresponding author.
