# Supplementary material for: What works in 21st century skills education in sub-Saharan Africa: a systematic review
Source: Front Psychol. 2025 Nov 10;16:1619154. doi: 10.3389/fpsyg.2025.1619154 (PMC12641231; doi:10.3389/fpsyg.2025.1619154)
Supplement: Supplementary file 1 [file Data_Sheet_1.docx]

What Works in 21^st^ Century Skills Education in Sub-Saharan Africa: A Systematic Review

List of Selected Articles for Analysis

1. Ambelu, A., Mulu, T., Seyoum, A., Ayalew, L., & Hildrew, S. (2019). Resilience dynamics after interventions made among school children of rural Ethiopia. *Heliyon*, *5*, e01464. https://doi.org/10.1016/j.heliyon.2019.e01464
2. Ashraf, N., Bau, N., Low, C., & Mcginn, K. (2020). Negotiating a better future: How interpersonal skills facilitate inter-generational investment. *The Quarterly Journal of Economics*, *135*(2), 1095–1151. https://doi.org/10.1093/qje/qjz039
3. Austrian, K., Soler-Hampejsek, E., Behrman, J. R., Digitale, J., Jackson Hachonda, N., Bweupe, M., & Hewett, P. C. (2020). The impact of the Adolescent Girls Empowerment Program (AGEP) on short and long term social, economic, education and fertility outcomes: A cluster randomized controlled trial in Zambia. *BMC Public Health*, *20*(1), 349. https://doi.org/10.1186/s12889-020-08468-0
4. Austrian, K., Soler-Hampejsek, E., Kangwana, B., Wado, Y. D., Abuya, B., & Maluccio, J. A. (2021). Impacts of two-year multisectoral cash plus programs on young adolescent girls’ education, health and economic outcomes: Adolescent Girls Initiative-Kenya (AGI-K) randomized trial. *BMC Public Health*, *21*(1), 2159. https://doi.org/10.1186/s12889-021-12224-3
5. Berger, R., Benatov, J., Cuadros, R., VanNattan, J., & Gelkopf, M. (2018). Enhancing resiliency and promoting prosocial behavior among Tanzanian primary-school students: A school-based intervention. *Transcultural Psychiatry*, *55*(6), 821–845. https://doi.org/10.1177/1363461518793749
6. Cherewick, M., Lebu, S., Su, C., Richards, L., Njau, P. F., & Dahl, R. E. (2021). Promoting gender equity in very young adolescents: Targeting a window of opportunity for social emotional learning and identity development. *BMC Public Health*, *21*(1), 2299. https://doi.org/10.1186/s12889-021-12278-3
7. Harding, R., Wei, G., Gwyther, L., & Miti, E. (2019). Improving psychological outcomes for orphans living with HIV in Tanzania through a novel intervention to improve resilience: Findings from a pilot RCT. *AIDS Care - Psychological and Socio-Medical Aspects of AIDS/HIV*, *31*(3), 340–348. https://doi.org/10.1080/09540121.2018.1533630
8. Hermosilla, S., Metzler, J., Savage, K., Musa, M., & Ager, A. (2019). Child friendly spaces impact across five humanitarian settings: A meta-analysis. *BMC Public Health*, *19*(1), 576. https://doi.org/10.1186/s12889-019-6939-2
9. Hosaka, K. R. J., Mmbaga, B. T., Gallis, J. A., & Dow, D. E. (2021). Feasibility and acceptability of a peer youth led curriculum to improve HIV knowledge in Northern Tanzania: Resilience and intervention experience from the perspective of peer leaders. *BMC Public Health*, *21*(1), 1925. https://doi.org/10.1186/s12889-021-11876-5
10. Ismayilova, L., Karimli, L., Sanson, J., Gaveras, E., Nanema, R., Tô-Camier, A., & Chaffin, J. (2018). Improving mental health among ultra-poor children: Two-year outcomes of a cluster-randomized trial in Burkina Faso. *Social Science & Medicine*, *208*, 180–189. https://doi.org/10.1016/j.socscimed.2018.04.022
11. Kachingwe, M., Chikowe, I., van der Haar, L., & Dzabala, N. (2021). Assessing the Impact of an intervention project by the Young women’s Christian Association of Malawi on psychosocial well-being of adolescent mothers and their children in Malawi. *Frontiers in Public Health*, *9*(585517). https://doi.org/10.3389/fpubh.2021.585517
12. Kibga, E. S., Sentongo, J., & Gakuba, E. (2021). Effectiveness of hands-on activities to develop chemistry learners’ curiosity in community secondary schools in Tanzania. *Journal of Turkish Science Education*, *18*(4), 605–621. https://doi.org/10.36681/tused.2021.93
13. McMullen, J. D., & McMullen, N. (2018). Evaluation of a teacher-led, life-skills intervention for secondary school students in Uganda. *Social Science and Medicine*, *217*, 10–17. https://doi.org/10.1016/j.socscimed.2018.09.041
14. Merrill, K. G., Merrill, J. C., Hershow, R. B., Barkley, C., Rakosa, B., DeCelles, J., & Harrison, A. (2018). Linking at-risk South African girls to sexual violence and reproductive health services: A mixed-methods assessment of a soccer-based HIV prevention program and pilot SMS campaign. *Evaluation and Program Planning*, *70*, 12–24. https://doi.org/10.1016/j.evalprogplan.2018.04.010
15. Metzler, J., Jonfa, M., Savage, K., & Ager, A. (2021). Educational, psychosocial, and protection outcomes of child- and youth-focused programming with Somali refugees in Dollo Ado, Ethiopia. *Disasters*, *45*(1), 67–85. https://doi.org/10.1111/disa.12392
16. Metzler, J., Saw, T., Nono, D., Kadondi, A., Zhang, Y., Leu, C. S., Gabriel, A., Savage, K., & Landers, C. (2023). Improving adolescent mental health and protection in humanitarian settings: Longitudinal findings from a multi-arm randomized controlled trial of child-friendly spaces among South Sudanese refugees in Uganda. *Journal of Child Psychology and Psychiatry and Allied Disciplines*, *64*(6), 907–917. https://doi.org/10.1111/jcpp.13746
17. Millanzi, W. C., Kibusi, S. M., & Osaki, K. M. (2022). Effect of integrated reproductive health lesson materials in a problem-based pedagogy on soft skills for safe sexual behaviour among adolescents: A schoolbased randomized controlled trial in Tanzania. *PLoS ONE*, *17*(2), e0263431. https://doi.org/10.1371/journal.pone.0263431
18. Ndetei, D. M., Mutiso, V., Gitonga, I., Agudile, E., Tele, A., Birech, L., Musyimi, C., & McKenzie, K. (2019). World Health Organization life-skills training is efficacious in reducing youth self-report scores in primary school going children in Kenya. *Early Intervention in Psychiatry*, *13*(5), 1146–1154. https://doi.org/10.1111/eip.12745
19. Nwokedi, O. P., Okeibunor, N. B., Ugwuanyi, J. C., Nwokolo, P. N., Ugwuoke, J. C., & Gever, V. C. (2023). Comparative analysis of the effectiveness of interactive radio and interactive television instructions on improvement in life skills among out-of-school nomadic children in Northern Nigeria. *Information Development*, *39*(3), 512–523. https://doi.org/10.1177/02666669221104599
20. Olowokere, A. E., & Okanlawon, F. A. (2018). Improving vulnerable school children’s psychosocial health outcomes through resilience-based training and peer-support activities: A comparative prospective study. *Vulnerable Children and Youth Studies*, *13*(4), 291–304. https://doi.org/10.1080/17450128.2018.1499988
21. Packer, C., Ridgeway, K., Lenzi, R., González-Calvo, L., Moon, T. D., Green, A. F., & Burke, H. M. C. (2020). Hope, self-efficacy, and crushed dreams: Exploring how adolescent girls’ future aspirations relate to marriage and childbearing in rural Mozambique. *Journal of Adolescent Research*, *35*(5), 579–604. https://doi.org/10.1177/0743558419897385
22. Pleaner, M., Milford, C., Kutywayo, A., Naidoo, N., & Mullick, S. (2022). Sexual and reproductive health and rights knowledge, perceptions, and experiences of adolescent learners from three South African townships: Qualitative findings from the Girls Achieve Power (GAP Year) Trial. *Gates Open Research*, *6*(60). https://doi.org/10.12688/gatesopenres.13588.1
23. Sebatana, M. J., & Dudu, W. T. (2022). Reality or mirage: Enhancing 21st-century skills through problem-based learning while teaching Particulate Nature of Matter. *International Journal of Science and Mathematics Education*, *20*(5), 963–980. https://doi.org/10.1007/s10763-021-10206-w
24. Stark, L., Asghar, K., Seff, I., Yu, G., Tesfay Gessesse, T., Ward, L., Assazenew Baysa, A., Neiman, A., & Falb, K. L. (2018). Preventing violence against refugee adolescent girls: Findings from a cluster randomised controlled trial in Ethiopia. *BMJ Global Health*, *3*(5), e000825. https://doi.org/10.1136/bmjgh-2018-000825
25. Stark, L., Seff, I., Asghar, K., Roth, D., Bakamore, T., MacRae, M., D’Andon, C. F., & Falb, K. L. (2018). Building caregivers’ emotional, parental and social support skills to prevent violence against adolescent girls: Findings from a cluster randomised controlled trial in Democratic Republic of Congo. *BMJ Global Health*, *3*(5), e000824. https://doi.org/10.1136/bmjgh-2018-000824
26. Torrente, C., Aber, J. L., Starkey, L., Johnston, B., Shivshanker, A., Weisenhorn, N., Annan, J., Seidman, E., Wolf, S., & Tubbs Dolan, C. (2019). Improving primary education in the Democratic Republic of the Congo: End-line results of a cluster-randomized wait-list controlled trial of learning in a healing classroom. *Journal of Research on Educational Effectiveness*, *12*(3), 413–447. https://doi.org/10.1080/19345747.2018.1561963
27. Willis, N., Milanzi, A., Mawodzeke, M., Dziwa, C., Armstrong, A., Yekeye, I., Mtshali, P., & James, V. (2019). Effectiveness of community adolescent treatment supporters (CATS) interventions in improving linkage and retention in care, adherence to ART and psychosocial well-being: A randomised trial among adolescents living with HIV in rural Zimbabwe. *BMC Public Health*, *19*(1), 117. https://doi.org/10.1186/s12889-019-6447-4
